# Reevaluating Patient Eligibility for Inotuzumab Ozogamicin Based on CD22 Expression: Is Dim Expression Sufficient?

**DOI:** 10.3390/curroncol28010027

**Published:** 2020-12-31

**Authors:** Warren Fingrut, Wendy Davis, Eric McGinnis, Karen Dallas, Khaled Ramadan, Hayley Merkeley, Heather Leitch, Yasser Abou Mourad, Ryan D. Cassaday, Camilla Ross, Chantal Léger

**Affiliations:** 1 Faculty of Medicine, University of British Columbia, Vancouver, BC V6T 1Z4, Canada; wendy.davis@alumni.ubc.ca (W.D.); eric.mcginnis@alumni.ubc.ca (E.M.); KDallas@providencehealth.bc.ca (K.D.); dr.ramadan@providencehematology.com (K.R.); hmerkeley@providencehematology.com (H.M.); drhleitch@providencehematology.com (H.L.); ymourad@bccancer.bc.ca (Y.A.M.); cross@providencehematology.com (C.R.); cleger@providencehematology.com (C.L.); 2 Department of Hematology, St. Paul’s Hospital, Providence Health Care, Vancouver, BC V6Z 1Y6, Canada; 3 Department of Pathology and Laboratory Medicine, Providence Health Care, Vancouver, BC V6Z 1Y6, Canada; 4 Leukemia/BMT Program of British Columbia, Vancouver General Hospital, Vancouver, BC V5Z 1M9, Canada; 5 British Columbia Cancer Agency, Vancouver, BC V5Z 4E6, Canada; 6 Department of Medicine, University of Washington, Seattle, WA 98195, USA; cassaday@seattlecca.org; 7 Clinical Research Division, Fred Hutchinson Cancer Research Center, Seattle, WA 19024, USA

**Keywords:** inotuzumab ozogamicin, cd22, acute lymphoblastic leukemia, flow cytometry, immunotherapy

## Abstract

Salvage options for patients with relapsed B-cell acute lymphoblastic leukemia (B-ALL) include inotuzumab ozogamicin (InO), a recombinant, humanized anti-CD22 monoclonal antibody conjugated to the cytotoxic antibiotic calicheamicin. However, the benefit of InO in patients with dim CD22 expression remains unclear. We present a case of a patient with B-ALL who responded to InO despite only dim surface expression of CD22 by flow cytometry, achieving a survival benefit concordant with that reported in the literature and maintaining a good quality of life as a transfusion-independent outpatient. Our observation has broad relevance to clinicians who manage patients with B-ALL who are candidates for InO.

## 1. Introduction

Salvage options for patients with relapsed B-cell acute lymphoblastic leukemia (B-ALL) include inotuzumab ozogamicin (InO), a recombinant, humanized anti-CD22 monoclonal antibody conjugated to the cytotoxic antibiotic calicheamicin. CD22 is expressed by more than 90% of B-cell blasts in nearly all patients with B-ALL [1]. However, the benefit of InO in patients with dim CD22 expression remains unclear.

## 2. The Case

**Figure 1:**
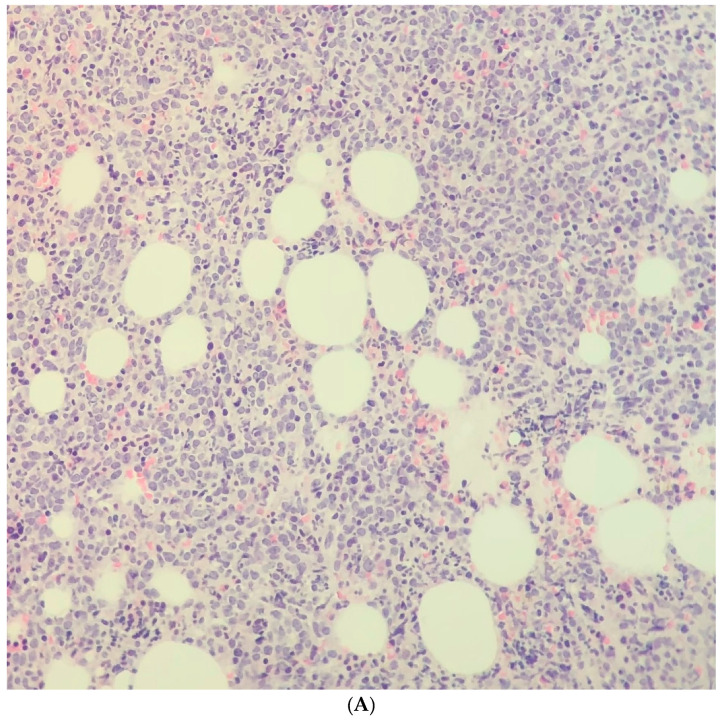

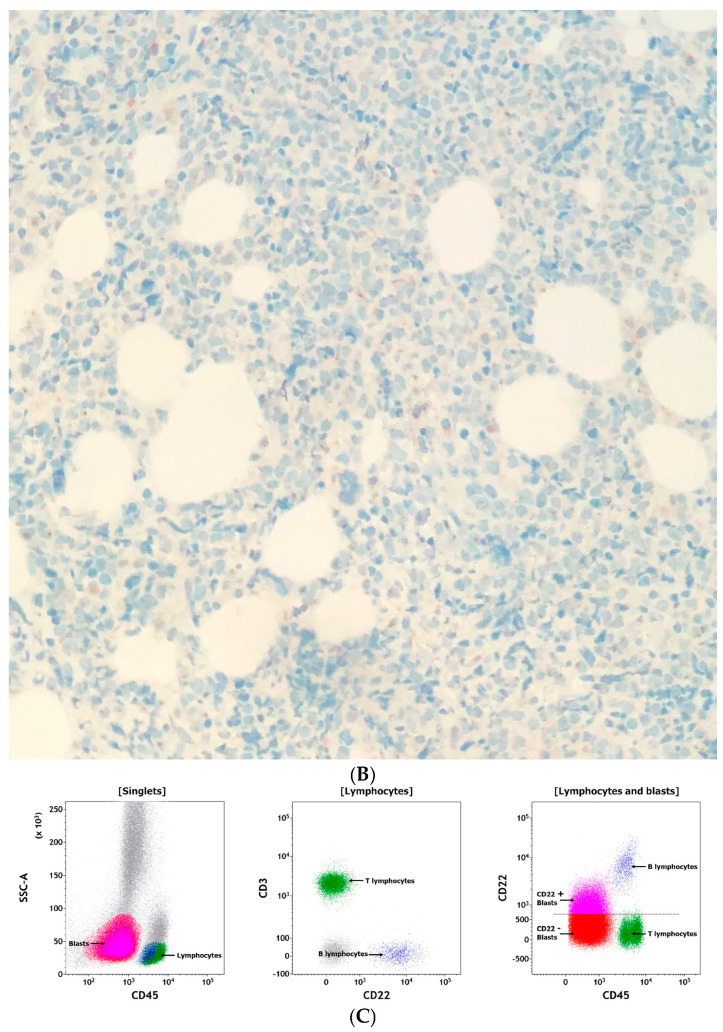
(**A**) Representative photomicrograph of the bone marrow biopsy diagnostic for relapse of B-ALL in the outlined patient case. The marrow space is packed by sheets of medium sized blasts and few interspersed hematopoietic cells (hematoxylin and eosin stain, 40× objective). (**B**) Representative photomicrograph of the immunohistochemical stain of marrow for CD22 in the outlined patient case (CD22 positive cells stain brown; 40× objective). (**C**) Flow cytometry of peripheral blood at time of relapse of B-cell acute lymphoblastic leukemia (gated populations displayed are indicated above panels). Left panel: CD45 versus side scatter plot showing CD45-dim blasts and CD45-bright lymphocytes. Middle panel: Normal B and T lymphocytes are indicated. Right panel: Blast CD22 expression compared to normal B and T lymphocytes. The patient’s T lymphocytes were used as an internal negative control for CD22 expression (threshold for blast positivity is indicated by the dashed line).

**Figure 2:**
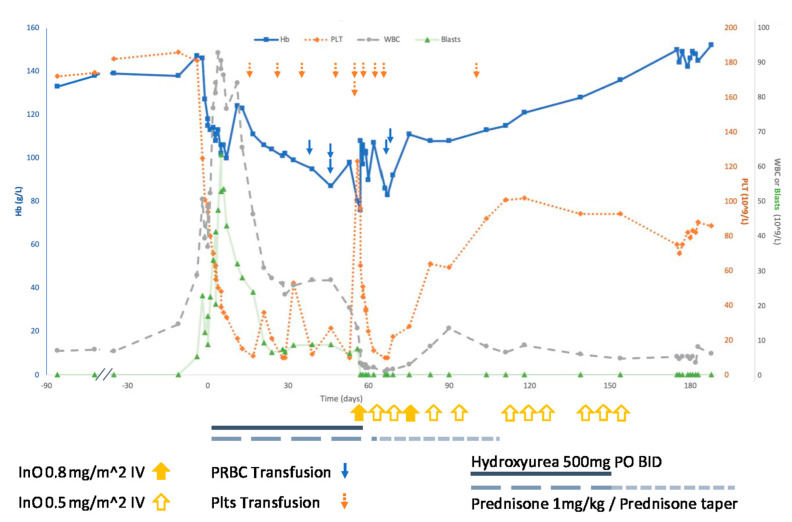
Serum hemoglobin (in g/L, primary *y* axis, squares with thick solid trend line), platelets (in platelets ×10^9^/L, secondary *y* axis, diamonds with dotted trend line), and white blood cells or blasts (in cells ×10^9^/L, tertiary *y* axis, white blood cells as circles with dashed trend line and blasts as triangles with thin solid line) shown longitudinally over time (days following diagnosis of relapse) for the outlined patient case. Treatments with (1) inotuzumab ozogamicin (InO) are shown as upward facing arrows along the *x* axis (solid: 0.8 mg/m^2^; hollow: 0.5 mg/m^2^); (2) hydroxyurea (500 mg PO BID) are shown below the *x*-axis as a solid black line); and (3) prednisone are shown below the *x*-axis as dashed grey line (large dashes: 1 mg/kg; small dashes: taper to off). Packed red blood cell transfusions are shown along the HB trend line as solid downward facing arrows, and platelet transfusions are shown at the top the graph as dashed downward facing arrows. Note: the dose of platelets transfused on day + 105 was ordered without an indication.

A 77-year-old woman at our center presented with a third relapse of Philadelphia-positive B-ALL after 15 months of treatment with tyrosine kinase inhibitors (7 months on imatinib, 3 on dasatinib, and 5 on ponatinib) initiated after intolerance to vincristine. At relapse, bone marrow biopsy showed 90% marrow cellularity, mostly B-ALL blasts (Figure 1A). BCR-ABL1 p210 transcript level was 38% (log < −0.42), increased from <0.01% (log < −4.0) five months prior. Molecular testing revealed T315I and Y253H BCR-ABL1 kinase domain mutations. White blood cell count was 50.6 × 109/L (45% blasts), and the patient was started on prednisone (1 mg/kg PO daily) and hydroxyurea (500 mg PO BID) to prevent hyperleukocytosis. Immunohistochemical staining of the marrow for CD22 showed no expression in the blasts (clone: SP104; Mayo Clinic Laboratories; Figure 1B). Flow cytometry performed on the peripheral blood using a panel designed for acute leukemia immunophenotyping identified a large population of blasts with an immunophenotype typical of B lymphoblastic leukemia (including dim expression of CD45 with low to intermediate side light scatter; expression of CD34, HLADR, CD19, CD20, and bright CD10; aberrant expression of CD56 and dim CD33; and no significant expression of other antigens typically associated with myeloid or T lymphoid differentiation). Notably, only 23% of blasts were positive for CD22, defined as fluorescence intensity greater than T lymphocytes, an internal negative control (BD Biosciences; fluorophore: PerCPCy5.5; clone: HIB22; Figure 1C). Nevertheless, the decision was made together with the patient to proceed with InO as salvage therapy. She received two cycles of 0.8 mg/m^2^ during week one, then 0.5 mg/m^2^ during weeks two and three, followed by two cycles of 0.5 mg/m^2^ weekly for three weeks. One day following the first dose of InO, the circulating blast count decreased to <0.1 × 10^9^/L from a prior plateau of 7−8 × 10^9^/L (Figure 2). Hydroxyurea was stopped and prednisone tapered to off. She achieved transfusion-independence with normalization of the hemoglobin and an increase in her platelet counts to 102 × 10^9^/L, whereas she had required five units of packed red blood cells and nine adult doses of platelets in the preceding two months (Figure 2). She tolerated InO without toxicity and remained an outpatient during treatment. After the fourth cycle, she presented with a one-week history of confusion and weakness. MRI of the brain demonstrated leptomeningeal involvement; flow cytometry of cerebrospinal fluid demonstrated blasts diagnostic of an isolated CNS relapse of previously diagnosed B-ALL, though insufficient sample was collected to test for CD22 expression. InO was discontinued and she was transitioned to comfort care.

## 3. Discussion

In our patient, despite only dim expression of CD22 on circulating blasts by flow cytometry, a survival benefit was achieved from InO concordant to that reported in the literature [2,3], and the patient enjoyed a good quality of life as a transfusion-independent outpatient without treatment-related toxicity. Limitations of interpretation of her course include the lack of marrow biopsy post-InO to formally assess response, the lack of flow cytometry results at time of relapse, and the subjective nature inherent in quantifying CD22 surface expression via flow cytometry.

There are several possibilities that may explain the patient’s clinical response to InO despite only dim expression of CD22 on circulating blasts by flow cytometry. CD22 is comprised of seven Ig-like domains that provide a number of membrane-distal epitopes that can be recognized by distinct monoclonal antibodies [4]. The G544 moiety of InO targets epitope A, which is located in the first N-terminal domain of CD22 [5]; however, the anti-CD22 antibody used in our center for flow cytometry (HIB22) binds two N-terminal domains of CD22, and the antibody used for immunohistochemistry (SP104) targets the C-terminal domain [6,7]. It is possible that the number of InO epitopes on the patient’s circulating blasts may not be concordant with the CD22 expression identified by flow cytometry or immunohistochemistry; both tests use different antibody clones, with different epitope targets than that of the G544 moiety of InO. Possible mechanisms of discordance include a mutation altering an epitope or expression of a truncated CD22 molecule missing epitopes. Furthermore, although the test principles between flow cytometry and immunohistochemistry are similar, laboratory processing differs substantially and could account for why CD22 expression was negative by immunohistochemistry but dim positive by flow cytometry. A possible explanation for the discrepancy between CD22 expression and InO response is that minimal expression of CD22 on blasts might be sufficient for InO to be internalized and deliver the calicheamicin payload causing apoptosis.

This case is the first to our knowledge that shows evidence of a response to InO in a patient with <30% CD22 expression on circulating blasts. In the INO-VATE randomized controlled trial (RCT), which compared InO to standard of care in patients with relapsed/refractory B-ALL, CD22 positivity was an inclusion criterion [3]. However, out of 164 patients randomized to receive InO, 30 had a CD22 expression of 70–90%, and a further 5 had a CD22 of <70% (of whom 3 had a complete remission and 2 achieved minimal residual disease-negative status). In a subgroup analysis comparing the efficacy of InO in patients with baseline leukemic blast CD22 positivity of ≥90% vs. <90%, a small but not statistically significant decrease in efficacy was seen when <90% of blasts were positive for CD22 [8]. Similar results were seen in a subgroup analysis of an RCT comparing InO + rituximab vs. chemotherapy + rituximab in patients with relapsed/refractory B-cell non-Hodgkin’s lymphoma [9]. Here, the group of patients receiving InO who had higher lymphoma cell CD22 expression (with a central laboratory-adjudicated immunohistochemical staining intensity H-score of ≥100/300, with score values ranging from 0 [no staining] to 300 [strong staining]) had nonsignificantly prolonged survival compared to those receiving InO with lower CD22 expression (H-score of < 100/300).

In our case, the patient developed isolated CNS relapse. A potential mechanism by which their disease relapsed is via a CD22 negative clone that was resistant to InO. It is also possible that the relapse occurred as a result of issues related to CNS penetration of InO. However, although data are limited with respect to CNS penetration of InO in humans, a preclinical in vivo study showed that InO protected against systemically disseminated B-ALL capable of invading the CNS and producing hind-limb paralysis in mice [10]. We also identified one case of a patient with relapsed/refractory lymphoid blast crisis of chronic myeloid leukemia involving the CNS who received intrathecal methotrexate plus systemic InO, leading to clearance of CNS disease and morphologic remission in the marrow [11]. Additional work is needed to better elucidate the mechanisms through which B-ALL relapses following therapy with InO.

## 4. Conclusions

In summary, we describe the case of a patient with B-ALL who responded to InO despite only dim surface expression of CD22 by flow cytometry, achieving a survival benefit concordant with that reported in the literature [2,3] and maintaining a good quality of life as a transfusion-independent outpatient. Our observation has broad relevance to clinicians who manage patients with B-ALL who are candidates for InO, not just in the relapsed/refractory setting, but in younger or fitter patients prior to allogeneic stem cell transplantation and in other settings where InO is used [9,11,12]. The patient’s course suggests that InO should be considered as a treatment option for patients with dim expression of CD22.
